# Case report: Ibogaine reduced severe neuropathic pain associated with a case of brachial plexus nerve root avulsion

**DOI:** 10.3389/fpain.2023.1256396

**Published:** 2023-09-01

**Authors:** Jonathan E. Dickinson, Jose Adalberto Dominguez Inzunza, Liliana Perez-Villa, Trevor G. Millar, Abhiram P. Pushparaj

**Affiliations:** ^1^ Board of Directors, Ambio Life Sciences, Vancouver, BC, Canada; ^2^ Medical Department, Ambio Life Sciences, Vancouver, BC, Canada; ^3^ Scientific Advisory, Ambio Life Sciences, Vancouver, BC, Canada; ^4^ Consulting Department, +ROI Regulatory Advisory, Toronto, ON, Canada

**Keywords:** brachial plexus nerve root avulsion, neuropathic pain, ibogaine, noribogaine, pain management, vehicular trauma

## Abstract

Brachial plexus nerve root avulsion results from complete separation of the nerve root from the spinal cord and is one of the most challenging types of neuropathic pain, coinciding with motor, sensory and autonomic deficits. The severe pain and typical impossibility of root reattachment often leads to requests for amputation. Ibogaine is an indole alkaloid producing psychoactive effects through reported actions upon multiple neurotransmitter systems, including NMDA, κ- and µ-opioid receptors and σ_2_ receptor sites, along with stimulation of neurotrophic factors GDNF and BDNF. In this case report we describe a 53-year-old male with two decades of severe intractable pain due to brachial plexus nerve root avulsion from vehicular trauma who was successfully treated with both high dose inpatient and low dose outpatient administrations of ibogaine. Though promising for future study, the adverse effects of high dose ibogaine administrations may limit tolerability of this saturation protocol to the most refractory cases.

## 1. Introduction

Brachial plexus nerve root avulsion is one of the most challenging types of neuropathic pain to treat effectively, and sometimes result in requests for amputation (1). Motosensory deficits can be significant in the case of root avulsion, wherein the nerve root separates completely from the spinal cord, typically due to vehicular trauma in young males (2). Reconstructive neurosurgery is the primary approach to preserving function in this type of severe injury, though with limited response (3).

Ibogaine is an indole alkaloid often referred to as oneirogenic (“dream-generating”) due to its complex effects on a range of neurotransmitter sites (4). It is most well-known as a putative therapeutic for mitigating withdrawal and cravings from opioids and other drugs (5), however it has also been studied experimentally for other conditions with a neurological basis (6). Ibogaine is administered orally as an encapsulated powder dosed by weight and is metabolized through the cytochrome 2D6 pathway, which converts ibogaine to its longer acting active metabolite noribogaine (7). Under normal conditions, ibogaine has a half-life of 7.5 h, and typical acute effects last for 18–36 h, while noribogaine has an estimated half-life of 28–49 h (8, 9).

Both ibogaine and its primary metabolite noribogaine are shown to stimulate the production of neurotrophic factors such as GDNF and BDNF (10, 11), which are targets for drug development in the treatment of Parkinson's and other neurodegenerative conditions (12). GDNF has been shown to prevent and reverse sensory abnormalities in neuropathic pain models (13). Ibogaine is also an NMDA receptor antagonist, which is consistent with ketamine (14, 15) and other drugs that have been investigated for neuropathic pain amelioration (16). Additionally, ibogaine is an agonist for σ receptors, with special affinity for σ_2_ (17). Compounds with similar σ receptor actions have been shown to attenuate neuropathic pain in mice (18–20).

There have previously been claims that ibogaine can reduce neuropathic pain (21), however, to our knowledge this is the first documented case report of its kind.

### 1.1. Case presentation

In March, 2022, a 53-year-old Caucasian male weighing 92.25 kg contacted an Ambio Life Sciences (“Ambio”) facility in Tijuana, Mexico inquiring about ibogaine treatment. 20 years prior, the patient had suffered a motorcycle accident resulting in brachial plexus nerve root avulsions on the right side C5, C6, C7. He subsequently underwent a number of reconstructive surgeries in order to avoid amputation of his right arm, including transfers of muscles and intercostal nerves to his biceps, transfer of left-side C7 nerves to his deltoids to prevent dislocation of shoulder muscles, tendon transfers to facilitate hand articulation, tricep reconstruction, and other fine-tuning surgeries. He had regained tactile sensation and partial motor functions. Upon intake at the medical facility, patient was he reported having experienced a high level of persistent toxic nerve pain since his injury, which had increased in intensity over the previous 10 years and was currently the worst he had experienced.

Before seeking out ibogaine the patient had exhausted other available treatment options, including pain medications like gabapentin, tramadol, oxycontin, and methadone, as well as tricyclic antidepressants, SSRIs, SSNRIs, and calcium channel anticonvulsants, all of which he stopped using in 2007, 14 years prior to intake. In the interim, he had found some short-term benefits during a clinical trial for rtMRI (22), as well as after stellate ganglion blocks on all 5 brachial plexus nerves, mirror box therapy (23, 24) mindfulness meditation training, experimental applications of cranial sacral therapy and experimental fetal stem-cell treatment. One option that he had yet to explore was a Dorsal Root Entry Zone lesion surgery, which has reported efficacy for similar injuries (25), but he was concerned about associated risks. He was curious about the potential of ibogaine for the treatment of neuropathic pain after reading anecdotal reports on the internet. However, his stated primary motivation was treatment of comorbid depression and other psychological factors related to his pain that had strongly affected his sleep and general mental health. These concerns aligned closely with Ambio's existing patient population.

At the time of intake, the patient reported high levels of pain on every scale assessed (please see Methodology below for details), which he described as typical of his usual pain experience. Subjectively, he described a constant sensation of “rawness, and burning pins and needles,” punctured by 4–10 episodes of breakthrough pain per hour while he was awake, which felt like “burning, searing, crushing, acid feel” throughout his right arm, or “like putting your arm in a boiling pot of acid.”

## 2. Treatment and qualitative observations

### 2.1. Methodology

The treatment is described in 4 distinct stages, which include 2 inpatient (T1, T2) and 2 subsequent outpatient events (O1, O2). T1 consisted of a standard “flood dose” of ibogaine hydrochloride (13.03 mg/kg) encapsulated in powder form, administered over the course of 2 h during an inpatient stay, while T2 dosing was conducted over a period of 7 days in cumulative doses (42.27 mg/kg total) with the intent of causing a “saturation” of ibogaine's primary metabolite noribogaine. During outpatient periods, the patient was provided with capsules of 10 mg of ibogaine hydrochloride and recorded his daily use in a pain journal. Doses began at 20 mg/day, and gradually increased as high as 250 mg/day in dialogue with the medical team, responding to pain severity. Please see Table 1 for a description of the dosing for each inpatient/outpatient period.

**Table 1:** Inpatient vs. outpatient treatment periods, durations, and dosing.

Period	Duration of dosing	Dosage
T1 Inpatient	1 day, “flood” dose	13.03 mg/kg
O1 Outpatient	17 days	10–200 mg per day
T2 Inpatient	7 days	42.27 mg/kg
O2 Outpatient	53 days	10–250 mg per day

At varying intervals, a series of questionnaires were administered, including the Douleur Neuropathique en 4 Questions (DN4) (26), Neuropathic Pain Symptom Inventory (NPSI) (27), Neuropathic Pain Questionnaire (NPQ) (28), Neuropathic Pain Scale (NPS) (27), and painDETECT (29). The NPQ, DN4, and painDETECT questionnaires are designed to be able to differentiate between neuropathic pain and non-neuropathic pain, while the NPS, NPQ, NPSI, and painDETECT instruments measure various characteristics of neuropathic pain (30). Several reviewers found the DN4 to have particular diagnostic validity (31, 32). In order to compare changes across measures, scores at each time point were covered to a *z*-score that was calculated in comparison with its respective measure (raw data table with conversions provided as Supplementary File). In addition to quantitative metrics, some scales request anecdotal descriptions, which are quoted in some instances.

During inpatient episodes patient underwent physical intake, full metabolic panel, and electrocardiogram. Vitals were monitored every 30 min during waking hours with 24-hour medical supervision. During treatment episodes greater than 5 mg/kg patient was under constant cardiac monitoring to screen for cardiac arrythmia including bradycardia. A patent-pending co-therapy protocol, including pre-treatment magnesium and vitamin infusions as well as post-treatment metabolic support was administered around both flood doses. Additional information was compiled from physician notes, electrocardiogram and cardiac monitor data, and semi-structured interview transcripts of the oneirogenic effects of ibogaine, which were collected during T1 and T2.

### 2.2. Observations

#### 2.2.1. T1

**Figure 1:**
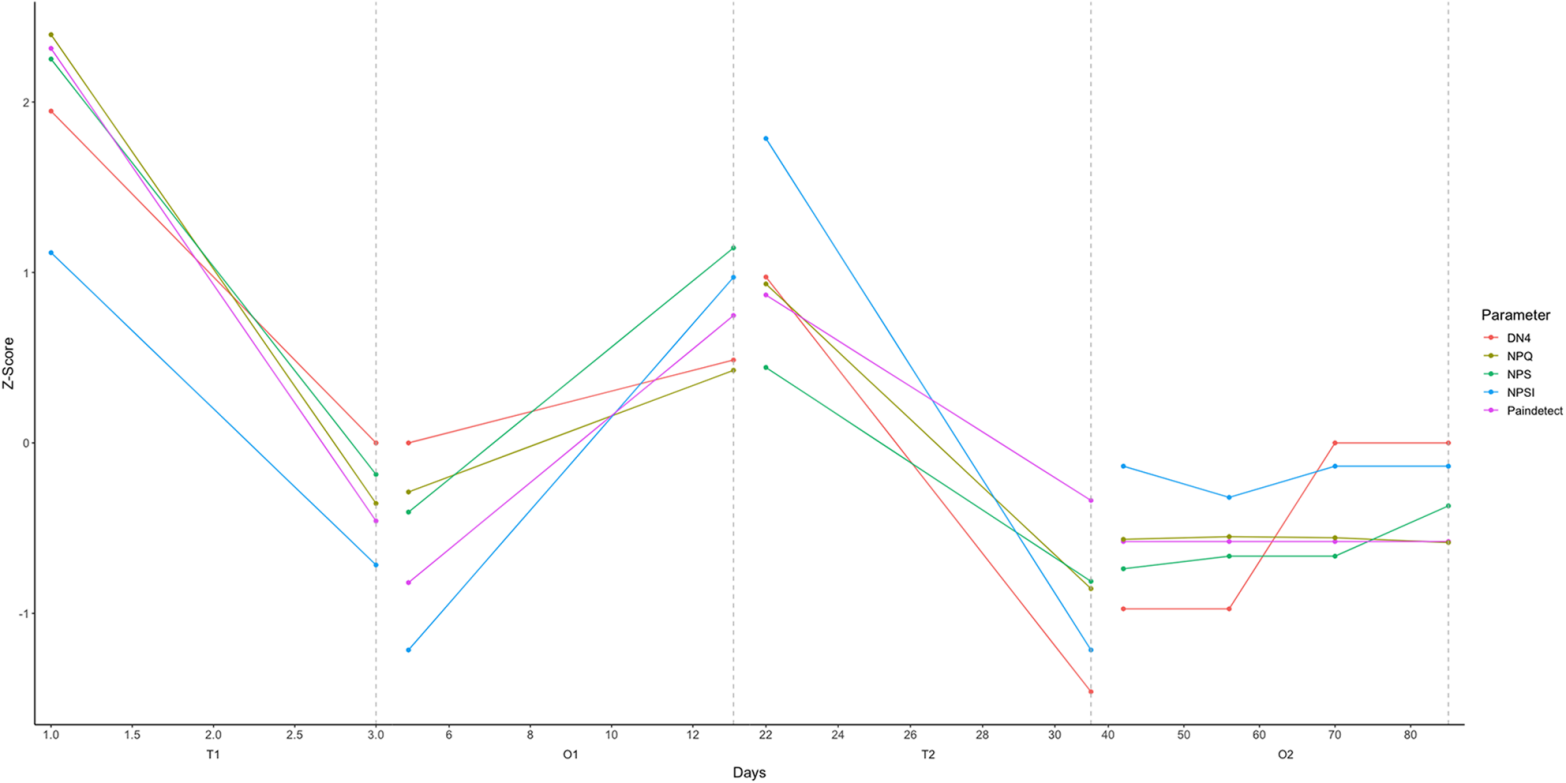
This graph shows changes to the subjective pain measures through each of the 4 treatment phases. *Z*-scores are derived for each of the scales individually, comparing the range of answers provided.

During T1 the patient reported strong oneirogenic effects that lasted roughly 12 h. This included visions of faces and other audio and visual distortions that seemed to appear in front of him in rapid succession. The overall theme of the experience was “like an unraveling of chaos,” that he described like “a ball of elastic bands if someone took a knife to it and it was chronically unraveling in a really weird way.” During periods where he had to focus on his body, such as getting up to go to the bathroom, he reflected that “the sensation of pain was not there.” Instead, “what was existing in my hand was just a sensation of numbness.” The patient reported marked reductions in pain experience for 2 days following treatment, amongst the most significant and most sustained he had experienced since his injury (Figure 1; end of T1).

#### 2.2.2. O1

O1 spanned 17 days following T1 discharge. Initially, the patient attempted doses of 10–50 mg, which lacked analgesic effect. Baseline pain levels steadily returned and breakthrough pain increased in intensity and frequency. The patient increased dosages to 150–200 mg/day. Although he noted improvement from pre-treatment levels in his pain journal, questionnaire scores returned back to 56.3% average across measures compared to baseline scores (Figure 1, end of O1). In this higher dose range, the patient complained of some uncomfortable side effects, such as lack of focus and productivity.

#### 2.2.3. T2

Patient was admitted for a period of 10 days for T2, during 7 of which ibogaine was administered. The dosing schedule, outlined in Table 2, was re-evaluated between each dose, through discussion between the patient, psychologist, attending physician, and medical director. Dosing was done one step at a time, attempting to strike a balance between tolerability and attempting to reach a high saturation of noribogaine. On Day 1 and 2, the patient reported “a good vibratory experience… it wasn’t obtrusive. It wasn’t overbearing.” He did experience visuals such as shadows and faces, forms approaching and talking to him, “like staring at an ocean wave… constantly changing,” with “more of a thrust behind it” each day. No cardiac morphology was noted.

**Table 2:** Daily dosing for T2 inpatient “saturation” period.

Day	Dosage (mg/kg)
1	2.71
2	5.96
3	18.97
4	0
5	3.79
6	5.42
7	5.42
Total	42.27

The morning of Day 3, 7.57 mg/kg was administered, and that same night another dose of 11.4 mg/kg split into 3 doses over the course of 1 h. This amounted to a total of 2,550 mg (27.7 mg/kg) over the course of 60 h. The patient described this experience as “much more intense than the days before it … The breath, the sound, the auditory, the active, everything was separating then reuniting and separating, reuniting.” Rather than appearing in front of him, things seemed to happen on “levels of expansive shifting fields,” which had greater depth of field than his previous experiences. Overall, the tone was “a realm darker, foreboding,” and he felt more nervous of the “bottom dropping out” from his mind. Unlike the flood dose in T1, he did feel some pain, although different from accustomed sensations. “Regularly, the charge is either a quick burn, or a fast burn, a single burn that has an electrical charge within it. This was just pure electrical charge that had much less variance to it.” Asymptomatic ventricular extrasystoles were observed, with no sign of hemodynamic instability.

Following a day of rest, additional dosages were administered on Days 5 through 7 to maintain noribogaine saturation. The patient reported that the pain was effectively eliminated by the end of the T2 period (Figure 1, end of T2). Only more subtle psychoactive effects were noted on these days, and sleep was reported to be improved.

#### 2.2.4. O2

Observations for O2 spanned 53 days following T2 discharge. The patient reported that dosages of 10–250 mg were able to manage his pain more effectively than any previous treatment. Questionnaire data showed pain levels at 21%–30% average across measures compared to baseline. Greatest benefit was reported for doses above 40 mg. Fluctuations of pain were also reported to be greatly reduced, even during weather changes, sickness including a confirmed case of COVID-19, and exposure to high altitude. These and similar experiences had been exacerbating factors in the past. On days when dosages were skipped or reduced, the patient reported return of baseline pain and increased frequency and intensity of breakthrough pain. Even at doses as small as 40 mg he continued to note the persistence of mild psychoactive effects throughout the day, which had unwanted effects on focus, such as fatigue, and limits to productivity caused by a propensity for introspection and some minor episodes of closed-eye visuals.

## 3. Quantitative outcomes

The results presented in Figure 1 demonstrate an acute analgesic effect of ibogaine in a case of severe treatment-resistant neuropathic pain, as well as a return of tactile sensation, results which were improved with increased and prolonged exposure to ibogaine and noribogaine. This effect was first demonstrated following a single flood dose (T1) with an immediate reduction of 72.2% across all subjective pain measures. The effect was most notable after the novel saturation protocol (T2), where we noted a 100% reduction on the DN4, and 91% average across all measures, compared to baseline. Following discharge in both instances (O1, O2), an analgesic effect was obtained using dosages of 40–250 mg/day, though this effect improved considerably following T2 saturation. During O2 we note reductions of 71%–80% average across pain measures when compared to baseline scores, which were sustained throughout the 2-month observation period during. Despite some persistent pain, the patient noted that it was easier to tolerate and to manage.

## 4. Discussion

This saturation protocol would not have been attempted were it not for the promising results from an initial flood dose, and the proximity of the two treatments likely had a cumulative effective. The patient received a total of 125.7 mg/kg over the entire 85-day course of treatment, and up to 19 mg/kg in a 24-hour period. No neurotoxic effects were observed at any point during the treatment. Previous literature described the destruction of Purkinje cells in animal models following ibogaine doses of 100 mg/kg (33). This has never been reported in humans, likely because the dose range is >4× the generally observed recommended 24-hour limit for cardiac safety and tolerability (34).

Cardiac abnormalities with ibogaine appear to be dose related, and this protocol required additional attention and care over a longer duration. Most concretely, ibogaine's effect of QT prolongation and risk of arrhythmia have resulted in documented cases of mortality and morbidity (35, 36). Ambio follows generally agreed upon clinical guidelines (34), which have been further elaborated by Ambio's medical team. During the course of this treatment, asymptomatic ventricular extrasystoles were observed at peak doses, with no additional cardiac abnormalities.

There are significant drawbacks to this approach to higher dose ibogaine saturation as a generalized treatment protocol. Most people find an ibogaine flood dose to be a challenging experience (5), and the saturation dosing method, the way it was approached, was considerably more intense and prolonged.

Further, the effects of the inpatient treatment alone may have been less substantial without follow-up dosages during the outpatient periods. The patient noted on several occasions, particularly during O2, that sometimes the psychoactive effects experienced at the dosages required to manage pain were undesirable, yet smaller dosages were less able to maintain the analgesic effect. We also note that the patient's pain condition was highly advanced, and theorize that benefits similar to those noted here may be easier to achieve in less severe cases.

During our data collection period, the patient did not take any medication other than those he was prescribed prior to arrival at the clinic and doses of said medications were not adjusted throughout data collection.

## 5. Conclusion

Brachial plexus nerve root avulsion results from complete separation of the nerve root from the spinal cord and is one of the most challenging types of neuropathic pain, coinciding with motor, sensory and autonomic deficits. The severe pain and typical impossibility of root reattachment often leads to requests for amputation. In the case of this patient, pain symptoms decreased by on average 50% over the follow-up period. Though this pain reduction has been sustained to date, the tolerability of the saturation protocol may limit its use to the most refractory patient population and should be studied further.

## Patient perspectives

The team of caregivers at Ambio Life Sciences couldn't have been more experienced, trustworthy, supportive, and outright professional. Ambio amply provided the grounded security needed to freely explore the uncharted depths of my being in hopes to free myself of this unwelcome parasitic neuropain. There's not enough space here to convey the full experience I had there. With absolute clarity (and amazement) I can say that my unrelenting pain was 100% gone for the first time in two decades. This overwhelming freedom carried on for a few amazing days afterwards, and I soon returned to continue what had been so brilliantly initiated. After my second round of treatments I was able to have windows of multiple days of total absence of pain. For the first time since my spine was injured I had actual numbness in my hand.This “new” numbness (from the original nerve damage) is what I should have been experiencing all along, if it had not been masked by the ceaseless searing all these years. This is no exaggeration. It was not wishful thinking. This incredible relief was extended in extremely tolerable degrees over periods of weeks as I experimented with microdosing levels of ibogaine to find the right sweet spot of administration. Occasional hints of pain would arise, far below the standard “base level” pain I was accustomed too. I could finally sleep, dream, hope, and plan again. Something I'd forgotten the genuine joy and importance of until it began to return to my life. This is a gift only few will understand the depths of, but I promise it is a gift that all in need should have immediately. I would still be medicating with ibogaine if it was legal where I live, so I'm planning to move to where it is legal very soon.

## Data Availability

The raw data supporting the conclusions of this article will be made available by the authors, without undue reservation.
