# Supplementary material for: Case report: Ibogaine reduced severe neuropathic pain associated with a case of brachial plexus nerve root avulsion
Source: Front Pain Res (Lausanne). 2023 Sep 1;4:1256396. doi: 10.3389/fpain.2023.1256396 (PMC10502345; doi:10.3389/fpain.2023.1256396)
Supplement: Supplementary file 2 [file Datasheet1.docx]

**PATIENT PERSPECTIVE:** *Ibogaine reduced severe neuropathic pain associated with a case of brachial plexus nerve root avulsion*

What I write here is purely in the interest of science and the importance of its latent potentials to heal those in desperate need. Without this hopelessness pervades and despair prevails unnecessarily.

Before my spinal cord injury at 34 years old, I thought I “knew pain”. I even used to compartmentalize difficult endeavors by reciting a favorite saying out loud “pain is only temporary” and I truly believed it. Over the years I’d broken over 26 bones from playing sports or dare-deviling as the youngest of 3 adventurous brothers might. At 19 I was accidentally shot through the center of my foot and waited 2 weeks before seeking medical attention due to the shame that followed from a childhood of intent respect for firearms’ dangers. At 21 I was randomly assaulted by a street gang, stabbed multiple times in the stomach, drove myself to a hospital while holding my intestines in, had 1/2 of my knifed liver removed, and I declined any painkillers when becoming conscious due to the cost of not having health insurance. At 24 I had bladder cancer, which required a check up “scoping” every 6 months for 10 years, with an instrument as fat as my pinky up the urethra of my penis, through the sphincters of my bladder, and up to my kidneys without the benefit of anesthetics. After my spinal injury at 34, in some twisted way to keep sane from the excruciating pain, I found myself whispering out loud to myself “I’ve been shot, stabbed, had cancer, broke 26 bones… I know pain, this isn’t fake, its real, I’m not insane”. This wasn’t imagined and I wasn’t ‘not tough enough’. This was an unforgiving pain that I never knew before.

At 54, out of sheer determined self-preservation, I’ve learned there are certain truths I rarely ever reveal to myself, because sometimes acknowledgement is a mirror too toxic to see. These truths are what follows. For the last 20 years my life has been governed and drastically limited by a relentless presence of chronic neuropathic pain. This pain has curtailed, disrupted, and/or destroyed every positive facet of my life, its richness, joy, and potentials. This “great isolator” is a psycho-spiritual-social cancer that devours any and all dreams, future plans, and hopes indiscriminately without warning or reason. While I have learned more from this pain than I’d ever imagined possible (including dangerous levels of; patience, curiosity, compassion, gratitude, willpower, determination, perseverance, empathy, etc) I’d still never wish its delivery on the worst of possible enemies. Countless months and years have fruitlessly been dedicated to its taming, befriending, and ultimate absolute eradication of. There comes a time when decisions must be made, and certain things must end, and this pain is one of those things.

The team of caregivers at Ambio Life Sciences couldn’t have been more experienced, trustworthy, supportive, and outright professional. Ambio amply provided the grounded security needed to freely explore the uncharted depths of my being in hopes of releasing myself from this unwelcomed parasitic companion of pain. There’s not enough space here to convey the depths of experience I had there, but with absolute clarity, and utter amazement, I can say that my unrelenting pain was 100% gone for the first time in two decades. This overwhelming freedom carried on for a few amazing days afterwards, and I soon returned to continue what had been so undeniably initiated. After my second round of treatments, I was able to have windows of multiple days of total absence of pain. For the first time since my spine was injured, along with three cervical nerve roots torn out from it, I had actual numbness in my hand. This ‘new’ sensory numbness was what I should have been experiencing all along, if it had not been masked by the ceaselessly searing, burning, and crushing sensations all these years. This is no exaggeration. It was not wishful thinking. This incredible relief was extended into extremely tolerable (yet ‘lesser’) degrees of relief over periods of weeks and weeks as I experimented with the microdosing levels of ibogaine to find the right ‘sweet spot’ of administration. Occasionally hints of pain would arise, but far below the standard ‘base level’ pain I was accustomed to, and the frequency was insignificant (a few times a day), especially compared to the previous 6-10 bursts per hour of mind crippling pain. I could finally sleep, dream, hope, and start to plan again. Something I’d forgotten the absolute joy and importance of until they began to reenter my life again.

When the ibogaine microdose sweet spot was achieved, my mental clarity, availability, and overall function was unlike any other medication I’d been treated with before. For the first five years post injury I unsuccessfully tried every pharmaceutical available for neuropathic pain that my pain specialists could offer. None of those medications achieved anywhere near the extreme relief I experienced from ibogaine, yet they all came with major mental and physical side effects, which I hopelessly endured for five years until I couldn’t any longer.  Ibogaine has no noticeable side effects, as long as the dosage was correct. In my experience, this medicine, ibogaine, is an incredibly important gift that all in need should have access to immediately. Unfortunately, ibogaine is not legal where I live in the USA, which is a crime in itself, so in order to get the life back again that ibogaine provided me, I will move to another country where access to this kind of pain relief is not a crime but a right. Thank you Ambio for helping forge this path to freedom from this ruthless maniacal burden.
